# Effects of fine motor-skill oriented sports games on core symptoms in children with autism: a randomized controlled trial

**DOI:** 10.1186/s13063-026-09651-1

**Published:** 2026-03-21

**Authors:** Yue Song, Jia Guo, Fengjie Yang, Yazeng Wu, Ping Zhang, Qiusheng Chen

**Affiliations:** 1 Department of Orthopedics, Wuhan Children’s Hospital, Tongji Medical College, Huazhong University of Science & Technology, Wuhan, 430016 China; 2 Department of Medicine and Health, Sichuan University of Science and Technology, Chengdu, 620500 China; 3 Rehabilitation Medicine, Xiantao First People’s Hospital, Xiantao, 433000 China

**Keywords:** Autism spectrum disorder, Core symptoms, Fine motor function, Functional assessment, Sports games

## Abstract

**Aims:**

Based on the observed correlation between core symptoms and fine motor function in children with ASD, this study aimed to evaluate the therapeutic effect of a novel, fine motor task-oriented sports game intervention, compared to traditional SI.

**Methods:**

Forty-five children with ASD were randomly allocated to two groups. The experimental group received the fine motor-oriented sports game intervention, while the control group received traditional SI. Core symptoms and fine motor function were assessed using the ABC, CABS, CARS, and PDMS-FM scales at both baseline and post-intervention, with the ABC scale serving as the primary outcome measure.

**Results:**

In the randomized comparison, the experimental group showed superior outcomes to the control group post-intervention, with significantly greater reductions in ABC, CARS, and CABS scores and a greater increase in PDMS-FM score (all *P* < 0.05).

**Conclusions:**

The fine motor-oriented sports game intervention proved more effective than SI in ameliorating the core symptoms and improving fine motor function in children with ASD.

**Trial registration:**

The study has been registered at ChiCTR.org (ChiCTR2400086052) on 2024-06-24.

**Supplementary Information:**

The online version contains supplementary material available at 10.1186/s13063-026-09651-1.

## Introduction

Autism spectrum disorder (ASD) is a lifelong heterogeneous neurodevelopmental disorder, characterized by social communication deficits and restricted/repetitive behaviors [1]. Global ASD prevalence has risen steadily, likely due to revised diagnostic criteria, optimized screening tools, and increased public awareness—amplifying familial and societal burdens [2].

ASD’s etiology/pathogenesis remains unclear (no specific diagnostic biomarkers identified) [3, 4] with potential links to genetics, environment, immune dysregulation, and prenatal factors [5]. While clinical diagnosis relies on the criteria outlined in the DSM-5 [6, 7] inconsistent evaluation standards and ambiguous intervention pathways in practice pose significant challenges for clinicians and families. While no curative medications or universally effective therapies currently exist for autism spectrum disorder, early detection and intervention are crucial as they lead to significantly better outcomes; conversely, prognosis tends to worsen with increasing symptom severity and delayed intervention [8, 9]. Therefore, a comprehensive assessment of the child’s functional profile is essential for tailoring effective interventions.

ASD assessment tools are pivotal for clinical practice, and their selection depends on the study population. Given that motor impairments are common in children with ASD, this factor must be considered when choosing appropriate tools [10]. However, research focusing specifically on fine motor function remains scarce—a key gap that this study aims to address.

To ensure the methodological and design validity of the randomized experiment, we first conducted an exploratory, hypothesis-generating preliminary analysis based on relevant indicators to determine whether a close association exists between the core symptoms of ASD and fine motor function. For this purpose, we utilized the Fine Motor Subscale of the Autism Behavior Checklist (ABC), the Clancy Autism Behavior Scale (CABS), the Childhood Autism Rating Scale (CARS), and the Peabody Developmental Motor Scales – Fine Motor (PDMS-FM) to assess fine motor skills and core symptoms in children with autism. Based on our findings from these analyses, we selected fine motor skill-oriented sports games as the intervention approach, aiming to address the core shortcomings of traditional rehabilitation methods. Conventional interventions (such as occupational therapy and sensory integration training (SI)) often rely on external equipment for repetitive exercises, which struggle to sustain engagement among children with ASD—a population characterized by attention deficits and lack of motivation. In contrast, structured sports games inherently possess motivating properties, allowing the demands of fine motor training to be seamlessly integrated into fun and interactive gameplay. This intervention model, aligned with the interests of children with ASD, can effectively enhance compliance and rehabilitation outcomes, making it an ideal and highly promising alternative intervention strategy for this population [11].

Based on this rationale, we hypothesized that (i) fine motor skills are correlated with core ASD symptoms, and (ii) our novel play-based motor program would alleviate these core symptoms via the enhancement of fine motor control. We conducted a 12-week randomized controlled trial to test these hypotheses, aiming to inform more targeted ASD interventions.

## Materials and methods

### Study design

A single-center, single-blind, randomized controlled trial was conducted. Before random grouping, we collected baseline assessment data from the patients’ family members and rehabilitation therapists to preliminarily explore the association between core symptoms and fine motor function. Subsequently, participants were randomly assigned to either the experimental or the control group. The randomization schedule was generated by drawing lots and concealed using sequentially numbered, sealed envelopes. To maintain the single-blind setup for the child participants, all interventions (both experimental and control) were delivered one-on-one in independent treatment rooms following standardized procedures, ensuring that the children remained unaware of their group assignment. Following a three-month intervention period, a final assessment was conducted. The data were then analyzed to evaluate within-group (pre- vs. post-intervention) and between-group differences. This analysis aimed to determine the efficacy of fine motor function-oriented sports games in improving the core symptoms of children with ASD.

### Participants

A total of 45 children with ASD (9 girls, 36 boys), aged between 1 and 6 years, were recruited from pediatric rehabilitation centers in Hubei Province, China. The age range of 1–6 years was selected to target the critical early intervention window for ASD, where core symptoms are becoming established [12]. While developmental abilities vary across this span, the core motor and sensory integration deficits targeted by our interventions are relevant across these ages in a preschool context. Written informed consent was obtained from all parents or legal guardians. Inclusion criteria comprised the following: (1) a formal diagnosis of moderate-to-severe ASD according to DSM-5 criteria, and (2) provision of consent by a guardian. Exclusion criteria included the following: significant head trauma; co-occurring neurological (e.g., epilepsy) or psychiatric disorders; severe behavioral problems precluding intervention; physical disabilities limiting participation; and significant uncorrected sensory impairments.

The study protocol was registered with the Chinese Clinical Trial Registry (Registration Number: ChiCTR2400086052, https://trialsearch.who.int/Trial2.aspx?TrialID=ChiCTR2400086052) and was reviewed and approved by the Research Ethics Committee of the College of Sports Medicine, Wuhan Sports University (Approval Number: 2023053; Date: July 5, 2023). In addition to guardian consent, we obtained child assent and implemented a continuous monitoring protocol. Throughout the intervention, therapists used age-appropriate engagement and behavioral cues to assess each child's state. Any indication of distress or reluctance triggered a predefined response protocol, which included the use of calming strategies, activity adaptation, or immediate session termination, ensuring the highest priority was given to participant safety and well-being.

### Outcomes measures

#### Primary outcome

We selected the ABC as the primary outcome measure to evaluate the core symptoms of autism. The ABC, developed by Krug et al. in 1980, is a well-established screening tool comprising 57 items. Each item is rated on a 4-point Likert scale from 0 (“not at all a problem”) to 3 (“the problem is severe in degree”). The total score (sum of all items) and five subscale scores (Sensory Behavior, Social Relating, Body and Object Use, Language and Communication Skills, and Social and Adaptive Skills) are derived, with higher scores indicating greater symptom severity. The reliability and validity of the Chinese version of the ABC have been strongly supported in populations with ASD [13].

#### Secondary outcomes

The CABS is designed to assess behavioral problems associated with ASD and is completed by parents or guardians. It consists of 14 items, each rated according to three frequency levels: “Never” (0 points), “Occasionally” (1 point), and “Often” (2 points). Based on established cutoff scores of 14 and 21, the total score classifies children into one of three categories. A score of 14 or higher—accompanied by fewer than 3 “Never” responses and more than 6 “Often” responses—suggests a potential autism diagnosis. The CABS has demonstrated validity and is extensively employed in mainland China [14].

The CARS is a widely recommended diagnostic tool for distinguishing children with ASD from those with other developmental delays. It consists of 15 items assessing a range of functions, including social interaction, emotional response, adaptive behavior, communication, and cognitive functioning. Each item is scored on a four-point scale, with higher scores indicating more severe symptoms. The total score categorizes individuals into three severity levels: Minimal-to-No Symptoms of ASD; Mild-to-Moderate Symptoms; and Severe Symptoms of ASD. CARS has demonstrated good reliability and validity in both research and clinical practice [15].

In addition to the CABS and CARS, we selected the PDMS-FM as a secondary outcome measure to assess fine motor skills. For children aged 0–72 months, the PDMS is one of the most widely used motor development assessment tools and can evaluate both fine and gross motor skills [16]. The present study focuses on the PDMS-FM, which comprises two subtests: Grasping (assessing midline hand movements, active grasping, object transfer between hands, and fine pincer grasp) and Visual-Motor Integration (evaluating eye-hand coordination). Each item is scored according to a three-point protocol: 2 points for correct task performance, 1 point for partial performance, and 0 points if the task is not performed correctly. The sum of item scores yields the raw score, from which a standard score, percentile rank, age equivalent, and *z*-score can be derived. The raw score directly reflects the child’s observed motor performance. To allow comparison across ages, raw scores are converted into a standard score (mean=10) based on normative data, indicating the child’s relative standing among peers of the same age. Finally, the standard score is transformed into the fine motor quotient (mean=100), which provides an overall developmental index and allows a comprehensive judgment of whether a child’s fine motor development is within the normal range, advanced, or delayed.

### Intervention

#### Intervention procedures

The study was conducted between July 2022 and July 2023. Baseline data were collected from 45 children with ASD prior to the intervention. Assessments were administered as follows: the ABC by individuals who had lived with the child for at least 2 weeks; the CABS by teachers and caregivers; and the CARS and PDMS-FM by professional doctors or rehabilitation therapists through observation, interview, and testing. The same assessor evaluated the same child at both baseline and post-intervention to ensure internal consistency of the ratings. During the pre-trial design phase, we conducted a series of preliminary, hypothesis-generating analyses based on this baseline data and relevant literature to examine the relationship between core symptoms and fine motor skills. Subsequently, a randomized controlled trial was initiated. Participants were randomly assigned to one of two groups: an experimental group that received fine motor skills-oriented sports games training, or a control group that received traditional SI.

**Fig. 1:**
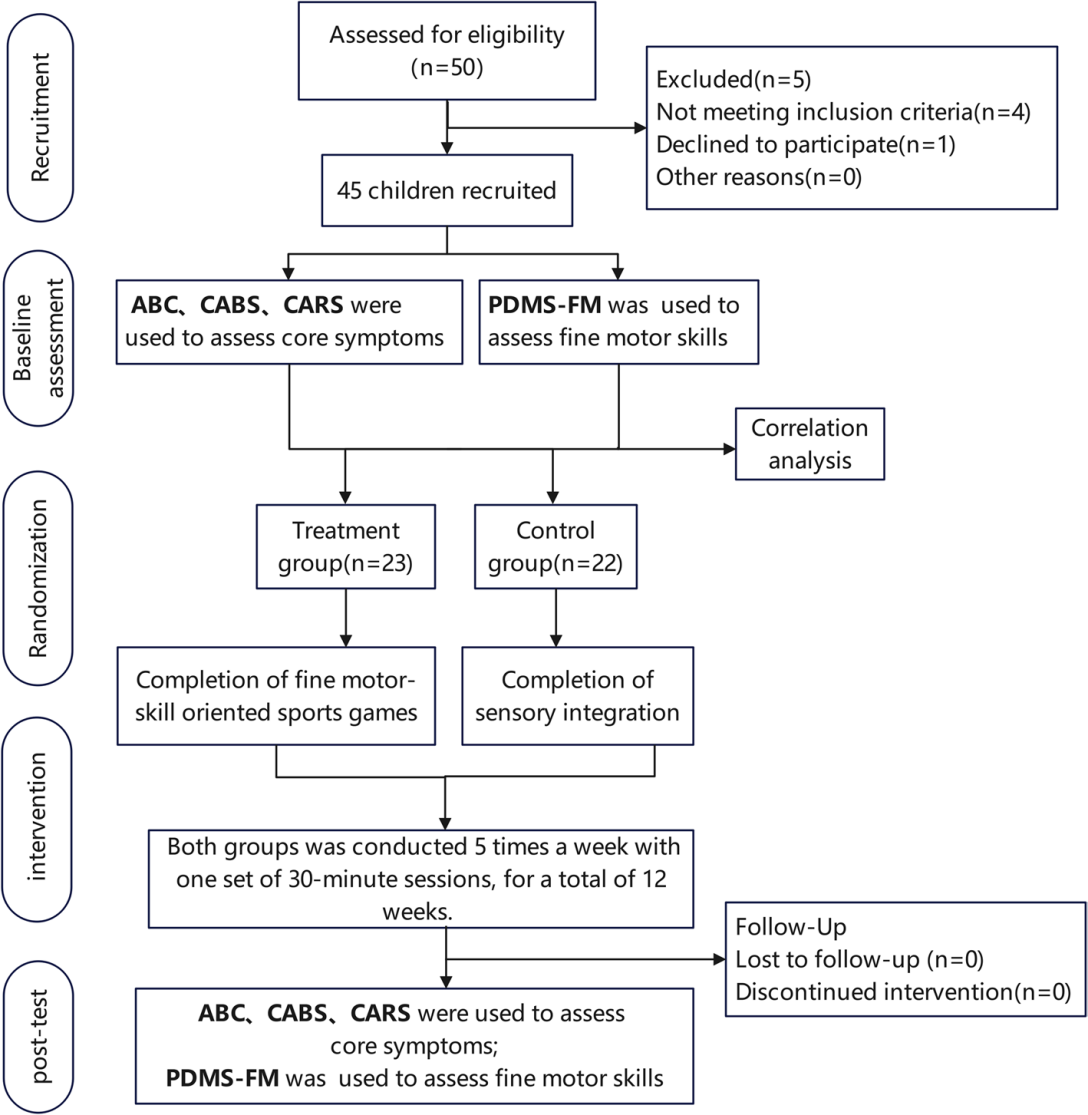
Study flow diagram

The allocation sequence was generated by a study statistician who was not involved in participant recruitment or intervention delivery, using a computer-based random number generator to create a sequence for 45 participants with a 1:1 allocation ratio between groups. This sequence predetermined the group assignment for each enrolled participant in consecutive order. To ensure allocation concealment, the group assignment corresponding to each random number was sealed in sequentially numbered, opaque envelopes prepared in advance by an independent research assistant. After obtaining written informed consent and confirming eligibility, the enrolling researcher opened the next consecutively numbered envelope in the participant’s presence. Participants assigned even numbers were allocated to the intervention group, and those with odd numbers to the control group. All interventions were administered separately according to group assignment. All participants were scheduled to undergo an intervention consisting of 5 sessions per week, each lasting 30 min, over a period of 12 weeks (a total of 60 sessions). To ensure that the total “dose” of intervention received by each participant was strictly consistent, we established a make-up class mechanism. If a child missed a training session due to objective reasons (such as illness, family affairs), the therapist would arrange additional time to make up for that session. Therefore, the total number of intervention sessions completed by all subjects was exactly the same. Following completion of the intervention, a post-intervention assessment was conducted in which all outcome measures were re-evaluated. It should be noted that participants remained blinded to their group allocation throughout the study; therapists were specifically assigned to only one intervention group to ensure specialization and consistency within each approach. A flow diagram of the study procedure is presented in Fig. 1.

#### Intervention in the treatment group

Participants allocated to the experimental group underwent a 12-week intervention consisting of three standardized sports games integrating sensory and motor training, designed for children aged 1–6 years. Each 30-min session was structured as follows: a 5-min warm-up, three 7-min activity blocks, and two 2-min rest intervals between blocks, resulting in 21 min of active training and 9 min dedicated to warm-up and rest. The games specifically targeted fine motor skills, including finger and wrist dexterity, aiming, catching, and hand–eye coordination. All Therapists followed a detailed manual with scripted instructions to ensure treatment fidelity. The intervention included a planned progression in difficulty every 4 weeks. For detailed information, please refer to the detailed intervention plan in the supplementary materials. The three games are described below:

- Pass and Catch: The child jumped freely on a mini-trampoline. A trained therapist, positioned at a standardized distance of 1.0 m, threw a soft foam ball toward the child’s torso. The child was instructed to catch the ball with both hands and throw it back to the therapist. The scripted instruction was: “[Child’s name], keep jumping! Catch the ball and throw it back to me.”
- Stringing Beads on the Balancing Board: The child stood on a balance board. While maintaining balance, the child was asked to string large wooden beads onto a shoelace. The therapist verbally encouraged weight shifting. The instruction was: “Try to stand steady. Pick up one bead and put it on the string. Now try the other side.”
- Sharp Eyes and Agile Hands: A target pole was placed 1.5 m in front of a therapy swing. The child, seated on the swing, was gently pushed by the therapist to initiate a predictable arc. At the forward peak of the swing, the child attempted to throw a rubber ring onto the target pole. The instruction was: “When you swing forward, try to ring the pole!”

#### Intervention in the control group

Both the intervention and control groups participated in structured, 30-min group sessions, held with the same frequency and total duration over the study period. The session structure—comprising a standardized warm-up, core activity blocks, and rest intervals—was identical for both groups to control for non-specific factors. A standardized protocol was followed to ensure consistency:

- Trampoline Training: The child performed continuous vertical jumps on a mini-trampoline, feet together. Following the standardized instruction and demonstration (“Jump up and down…landing with both feet together”). Progression involved adding knee tucks at Week 5 and coordinated arm swings from Week 9.
- Balancing Board Training: Lying prone on a balance board, the child rocked it side-to-side using core strength after the instruction, “Hold on tight…rock the board side to side like a seesaw.”The amplitude increased from Week 5, and from Week 9, the task was performed with eyes closed.
- Swing Play: The child sat on a therapy swing while the therapist applied a fixed rotation sequence, totaling 7–8 min per session. Rotation speed increased slightly from Week 5, and brief, unexpected pauses were introduced in the final 4 weeks to challenge postural adjustment.

#### Trial management and safety monitoring

This single-center trial was conducted at Wuhan Sport University. The PI and the trial coordinator were responsible for all aspects of the trial, including finalizing the protocol, obtaining ethical approvals, ensuring protocol adherence, and performing the final data analysis. Day-to-day operations were conducted by a team comprising: (1) trained therapists delivering the interventions; (2) blinded assessors conducting outcome evaluations; and (3) a coordinator managing recruitment and logistics. The team held weekly meetings with the PI to review progress and ensure fidelity.

Regarding oversight and safety monitoring, a formal independent Trial Steering Committee or Data and Safety Monitoring Board was not established, given the behavioral, non-invasive, and minimal-risk nature of the interventions. Oversight responsibilities were undertaken by the PI and the core research team. An active monitoring program was implemented, which included the following: (1) therapists using standardized logs to record participant tolerance and any signs of distress during each session; (2) systematic use of structured questionnaires in all assessment visits to interview parents/guardians about any emergent health or behavioral issues; and (3) documentation of all such events. Any serious adverse event (defined as an event leading to death, hospitalization, severe disability, or intervention cessation) would have been immediately reported to the institutional ethics committee that approved the study (Human Experiment Ethics Consideration Committee of the School of Sports Medicine, Wuhan Sport University). Overall trial conduct and safety data were reviewed by the PI and co-investigators during monthly project reviews.

#### Trial governance and protocol amendments

A formal procedure will be followed for any amendment to the study protocol. Proposed amendments, initiated by the PI, must be submitted in writing to the study sponsor and the approving Institutional Review Board (IRB) for scientific and ethical review. Implementation will only commence after written IRB approval is obtained. The PI will then notify all participating centers and investigators, providing the revised protocol and approval documentation. Any unintended deviations from the protocol will be recorded immediately using a Protocol Deviation Report Form, assessed for their impact on participant safety and study integrity, reported to the IRB as necessary, and retained in the trial master file.

### Sample size

The sample size calculation was based on the post-intervention ABC total scores expected for the two groups. With reference to a previous study [17], we assumed mean (SD) ABC scores of 66 (10.0) for the experimental group and 52 (10.0) for the control group. Using PASS software (version 15.0), a minimum sample size of 22 participants (11 per group) was determined to achieve a significance level of 5% and 90% power (*α* = 0.05, 1-*β* = 0.90). To account for a potential dropout rate of 20%, at least 28 participants were planned to be enrolled in the study.

### Statistical analysis

All statistical analyses were performed using SPSS software (version 25.0). To preliminarily examine the relationship between core autism symptoms and fine motor ability, bivariate scatter plots were generated. In these plots, the total scores from the core symptom assessment scales (ABC, CABS, and CARS) were treated as the independent variable (*X*-axis), and the PDMS-FM score was treated as the dependent variable (*Y*-axis). The resulting scatter plots revealed a tightly clustered distribution of data points, visually indicating a linear association between these variables and justifying further formal correlation analysis. Before conducting the primary and correlational analyses, we formally assessed the normality of all continuous variables. Given the sample size (*n* = 45), the Shapiro-Wilk test was appropriately employed. The results indicated that the distributions of the ABC (*W* = 0.96,* P* = 0.14), CABS (*W* = 0.96, *P* = 0.15), CARS (*W* = 0.98, *P* = 0.67), Grasping (*W* = 0.95, *P* = 0.06), Visual-Motor Integration (*W* = 0.95, *P* = 0.07), and FMQ (*W* = 0.98, *P* = 0.71) scores did not significantly deviate from normality. Consequently, the use of parametric tests for these variables was deemed justified. Descriptive data are presented as mean ± standard deviation. If the baseline levels are balanced across groups, an independent sample t-test is used for group comparison. However, when there are clinically relevant or statistically significant differences in the baseline between groups, to control the potential impact of this baseline imbalance on the post-intervention results, we use analysis of covariance for comparison. Specifically, the post-intervention scores are used as the dependent variable, the groups are used as the fixed factor, and the baseline scores are used as the covariate for the analysis. A paired-sample *t*-test was used to evaluate within-group differences between baseline and pre-test measurements. All participants randomized to either intervention or control groups were included in the intention-to-treat analysis, regardless of whether they completed the full intervention or received additional tutoring. Missing outcome data were imputed using the last observation carried forward method to maintain the comparability of groups established by randomization. To transparently quantify the magnitude of any baseline differences, we also calculated Cohen’s d, the mean difference, and its 95% confidence interval for all continuous variables. Statistical significance was set at *P* < 0.05. Figures were generated using GraphPad Prism 9.0.

## Results

### Preliminary exploration of between core symptoms and fine motor skills in children with ASD

**Fig. 2:**
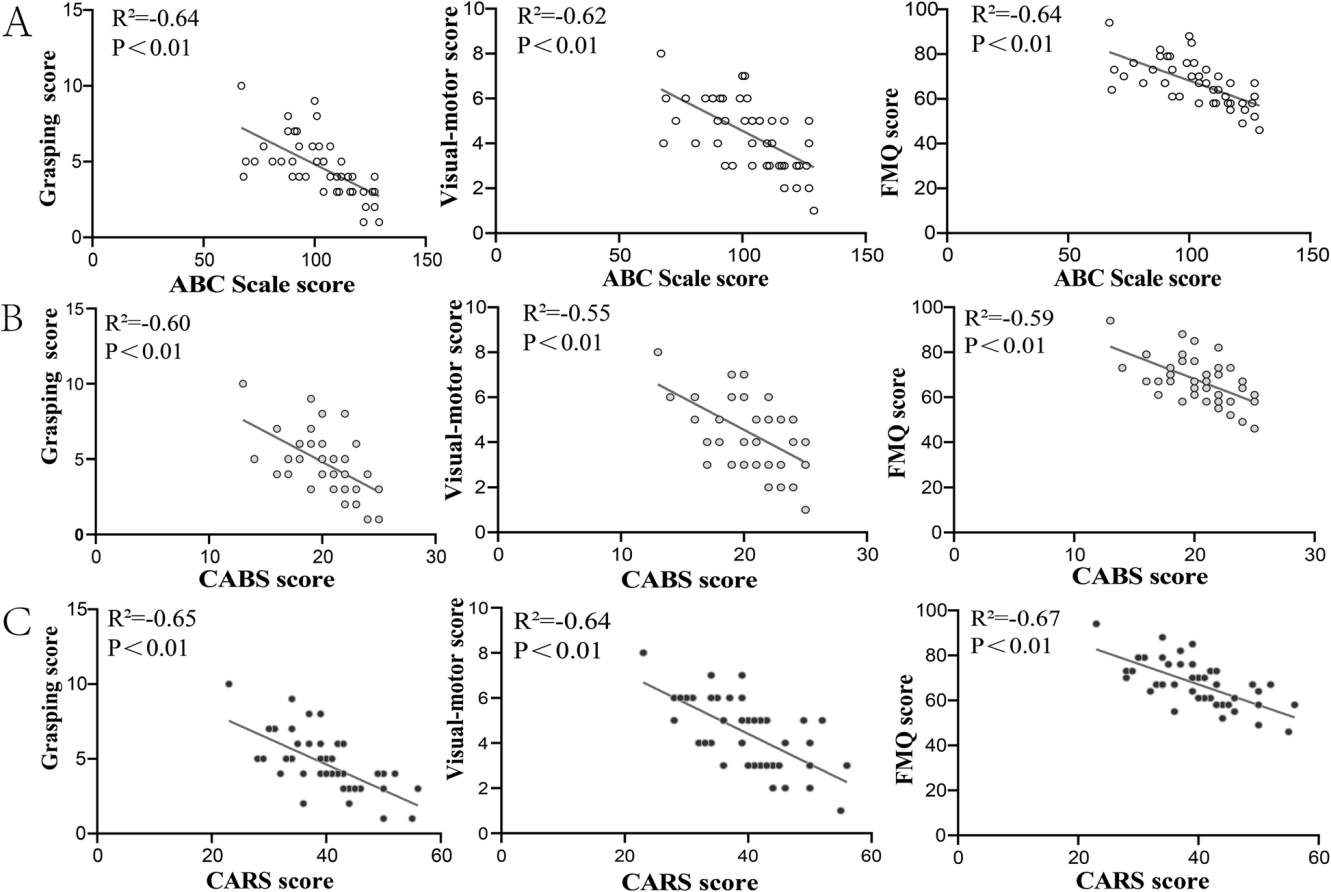
Preliminary exploration between core symptom and fine motor function. Note: **A** Simple linear regression between ABC scores (Autism Behavior Checklist; higher scores = more severe autism symptoms) and PDMS-FM indices (grasping, visual-motor integration, FMQ; higher scores = better performance). **B** Simple linear regression between CABS scores (Childhood Autism Behavior Scale; higher scores = more severe autism symptoms) and PDMS-FM indices (grasping, visual-motor integration, FMQ; higher scores = better performance). **C** Simple linear regression between CARS scores (Childhood Autism Rating Scale; higher scores = more severe autism symptoms) and PDMS-FM indices (grasping, visual-motor integration, FMQ; higher scores = better performance)

As shown in Fig. 2, significant negative correlations were found between the ABC scale and all measured fine motor domains: gripping ability (*r* = −0.64), visuomotor integration (*r* = −0.62), and fine motor development quotient (*r* = −0.64) (all *P* < 0.01). Similarly, the total CABS score also showed significant negative correlations with these fine motor skills (*r* = −0.60, −0.55, −0.59, respectively; all *P* < 0.01). Furthermore, the exploratory analysis revealed that significant negative correlations between the total CARS score and all three fine motor measures (*r* = −0.65, −0.64, −0.67, respectively; all *P* < 0.01).

### Baseline data

This study enrolled 45 participants, with no dropouts occurring throughout the trial. No serious adverse events were reported, nor were any other adverse events deemed related to the interventions. Table 1 presents the demographic information and baseline assessment scores for participants in both groups. A detailed comparison confirms that there were no significant differences between the treatment and control groups at baseline in terms of age, gender, core symptom severity, or fine motor function. All calculated Cohen’s *d* values were below 0.5, indicating small effect sizes and confirming that the groups were well-matched and comparable prior to the intervention.

**Table 1:** Baseline characteristics of participants in the control and treatment groups

Variables		Control	Treatment
*N*		22	23
Age		54.27 ± 13.96	50.87 ± 16.35
Gender	Male	17 (38%)	19 (42%)
	Female	5 (12%)	4 (8%)
ABC		105.55 ± 15.74	99.65 ± 17.98
CABS		20.86 ± 2.80	20.04 ± 3.04
CARS		41.77 ± 7.86	38.13 ± 6.63
Grasping		4.45 ± 1.90	4.83 ± 2.01
visual-motor		4.23 ± 1.63	4.61 ± 1.46
FMQ		66.05 ± 10.28	68.30 ± 10.27

Note: This table presents the baseline characteristics of participants in the control group and treatment group. Continuous variables are reported as mean ± standard deviation, and categorical variables (Gender) are reported as *n* (%). Comparisons between groups were conducted using independent-samples *t*-tests (for continuous variables) or chi-square tests (for Gender)

*Abbreviations*: *ABC* Autism Behavior Checklist, *CABS* Childhood Autism Behavior Scale, *CARS* Childhood Autism Rating Scale, *Grasping* Fine motor grasping skill score, *visual-motor *Visual-motor integration skill score, *FMQ* Fine Motor Quotient

### Comparisons of intra-group and inter-group differences in core symptoms and fine motor function

All outcome variables changed following the training sessions. In the treatment group, paired-sample *t*-tests revealed significant improvements from pre-test to post-test across all assessment measures (*P* < 0.05). Specifically, significant reductions were observed in the total ABC score (*t* = 12.83, *P* < 0.01), CABS score (*t* = 16.07, *P* < 0.01), and CARS score (*t* = 10.39, *P* < 0.01). These were accompanied by significant increases in grasping (*t* = −12.24, *P* < 0.01), visual-motor integration (*t* = −11.47, *P* < 0.01), and fine motor quotient (*t* = −17.43, *P* < 0.01). Similarly, in the control group, paired-sample t-tests also indicated significant improvements in several outcomes: total ABC score (*t* = 11.91, *P* < 0.01), CABS score (*t* = 2.19, *P* < 0.05), CARS score (*t* = 2.69, *P* < 0.01), visual-motor integration (*t* = −2.35, *P* < 0.05), and fine motor quotient (*t* = −2.85, *P* < 0.01). However, the improvement in grasping was not statistically significant (*t* = −1.82, *P* = 0.08). Although there was no statistically significant difference in the baseline ABC scores between the two groups, considering that there was approximately a 5-point difference in the baseline ABC scores between the experimental group and the control group, this difference might have clinical significance. Therefore, we used the ANCOVA model to analyze the ABC scores after the intervention, with the baseline score as the covariate (Table 2). The results showed that after adjusting for the baseline imbalance, the ABC scores of the fine motor game training group were still significantly lower than those of the traditional sensory integration training group, and the group difference was statistically significant (*F* = 27.59, *P* < 0.01, partial *η*^2^ = 0.40). Furthermore, independent sample *t*-tests for other indicators indicated that the treatment group showed significantly greater improvement than the control group in CABS score (*t* = −7.42, *P* < 0.01), CARS score (*t* = −7.05, *P* < 0.01), grasping (*t* = 3.25, *P* < 0.01), visual-motor integration (*t* = 2.71, *P* < 0.05), and fine motor quotient (*t* = 3.19, *P* < 0.01) (Table 3).

**Table 2:** ANCOVA analysis of the ABC scores of the experimental group and the control group after intervention

Group	Baseline (Mean ± SD)	Post-intervention (Mean ± SD)	Adjusted mean (95%CI)	*F*	*P*	Partial *η*^2^
Control (*n* = 22)	105.55 ± 15.74	95.23 ± 16.55	92.35 ± 1.28(89.76 ~ 94.94)	27.59	< 0.01	0.40
Study (*n* = 23)	99.65 ± 17.98	80.09 ± 17.83	82.84 ± 1.26(80.31 ~ 85.37)			

Note: For the ABC score, post-intervention comparisons were conducted using covariance analysis. This table. presents the adjusted mean (95% confidence interval), as well as the corresponding *F* value, *P* value, and effect size (partial *η*^2^)

**Table 3:** Pre-test and post-test comparison of core symptoms and fine motor function between the groups

Variables	Groups	*N*	Pre-test	Post-test	Dependent *T*	*p*	Cohen’s *d*	MD	95%CI	Independent *T*	*p*	Cohen’s *d*	MD	95%CI
ABC	Control	22	105.55 ± 15.74	95.23 ± 16.55	11.91	< 0.01	2.54	10.32	8.52 ~ 12.12	−2.95	< 0.01	0.88	−15.14	−25.49 to approximately −4.79
	Study	23	99.65 ± 17.98	80.09 ± 17.83	12.83	< 0.01	2.67	19.57	16.40 ~ 22.73					
CABS	Control	22	20.86 ± 2.80	20.23 ± 2.09	2.19	0.04	0.47	0.64	0.03 ~ 1.24	−7.42	< 0.01	2.21	−5.71	−7.26 to approximately −4.16
	Study	23	20.04 ± 3.04	14.52 ± 2.97	16.07	< 0.01	3.35	5.52	4.81 ~ 6.23					
CARS	Control	22	41.77 ± 7.86	40.64 ± 6.59	2.69	0.01	0.57	1.14	0.26 ~ 2.02	−7.05	< 0.01	2.10	−11.64	−14.97 to approximately −8.31
	Study	23	38.13 ± 6.63	29.00 ± 4.31	10.39	< 0.01	2.17	9.13	7.31 ~ 10.95					
Grasping	Control	22	4.45 ± 1.90	4.73 ± 1.88	−1.82	0.08	0.39	−0.27	−0.58 to approximately 0.04	3.25	< 0.01	0.97	1.75	0.67 ~ 2.84
	Study	23	4.83 ± 2.01	6.48 ± 1.73	−12.24	< 0.01	2.55	−1.65	−1.93 to approximately −1.37					
visual-motor	Control	22	4.23 ± 1.63	4.59 ± 1.40	−2.35	0.03	0.50	−0.36	−0.67 to approximately −0.04	2.71	< 0.05	0.81	1.19	0.31 ~ 2.08
	Study	23	4.61 ± 1.46	5.78 ± 1.54	−11.47	< 0.01	2.39	−1.17	−1.39 to approximately −0.96					
FMQ	Control	22	66.05 ± 10.28	67.95 ± 9.32	−2.85	0.01	0.61	−1.91	−3.30 to approximately −0.51	3.19	< 0.01	0.95	8.83	3.25 ~ 14.41
	Study	23	68.30 ± 10.27	76.78 ± 9.23	−17.43	< 0.01	3.63	−8.48	−9.49 to approximately −7.47					

Note: This table presents the pre-intervention and post-intervention scores of core symptoms (ABC, CABS, CARS) and fine motor function (Grasping, visual-motor integration, FMQ) for children with autism in the fine motor-oriented physical play group (Study) and the traditional SI group (Control). Differences in intervention efficacy between the two groups were compared using intra-group and inter-group *t*-tests, effect sizes and other relevant indicators

*Abbreviations*: *ABC* Autism Behavior Checklist, *CABS* Childhood Autism Behavior Scale, *CARS* Childhood Autism Rating Scale, *Grasping* fine motor grasping skill score, *visual-motor integration* visual-motor integration skill score, *FMQ* fine motor quotient, *MD* mean difference, *95%CI* 95% confidence interval

Column explanations: Dependent *T* = Within-group *t*-test (pre-test vs. post-test); Independent *T* = Between-group *t*-test (post-intervention scores: Control group vs. Study group)

### Intra-group and inter-group comparisons of total ABC score and ABC subscale scores

Paired samples *t*-tests were performed to evaluate the effects of the two interventions on the core symptoms of children with ASD (Table 4). In the treatment group, post-intervention scores for all outcome measures showed statistically significant improvement compared to baseline (all *P* < 0.01). In the control group, significant pre-post improvements were observed in the total ABC score (*t* = 11.91, *P* < 0.01), Sensory subscale (*t* = 13.03, *P* < 0.01), and Body and object use subscale (*t* = 8.89, *P* < 0.01). However, no significant changes were found in the Relating (*t* = 0.72, *P* = 0.48), Language (*t* = 2.03, *P* = 0.06), or Social and self-help (*t* = 0.65, *P* = 0.53) subscales. Between-group comparisons revealed that the treatment group demonstrated significantly greater improvement than the control group in the total ABC score (*t* = −2.95, *P* < 0.01) as well as in the Relating (*t* = −3.60, *P* < 0.01), Language (*t* = −2.54, *P* < 0.05), and Social and self-help (*t* = −2.23, *P* < 0.05) subscales. No significant between-group differences were observed for the Sensory (*t* = −0.37, *P* = 0.71) or Body and object use (*t* = −0.92, *P* = 0.36) subscales after the intervention.

**Table 4:** Pre-test and post-test comparisons of total ABC score and ABC subscale scores between groups

Variables	Groups	*N*	Pre-test	Post-test	Dependent *T*	*P*	Cohen’s *d*	MD	95%CI	Independent *T*	*P*	Cohen’s *d*	MD	95%CI
ABC	Control	22	105.55 ± 15.74	95.23 ± 16.55	11.91	< 0.01	2.54	10.32	8.52 ~ 12.12	−2.95	< 0.01	0.88	−15.14	−25.49 to approximately −4.79
	Study	23	99.65 ± 17.98	80.09 ± 17.83	12.83	< 0.01	2.67	19.57	16.40 ~ 22.73					
Sensory	Control	22	19.59 ± 4.13	15.00 ± 4.83	13.03	< 0.01	2.78	4.59	3.86 ~ 5.32	−0.37	0.71	0.11	−0.57	−3.63 to approximately 2.50
	Study	23	19.61 ± 5.26	14.43 ± 5.32	9.40	< 0.01	1.96	5.17	4.03 ~ 6.32					
Relating	Control	22	29.55 ± 5.89	29.41 ± 5.50	0.72	0.48	0.15	0.14	−0.26 to approximately 0.53	−3.60	< 0.01	1.07	−6.19	−9.66 to approximately −2.72
	Study	23	26.74 ± 4.65	23.22 ± 6.00	5.42	< 0.01	1.13	3.52	2.17 ~ 4.87					
Body and object use	Control	22	18.55 ± 8.24	13.55 ± 6.55	8.89	< 0.01	1.90	5.00	3.83 ~ 6.17	−0.92	0.36	0.27	−1.81	−5.77 to approximately 2.16
	Study	23	17.39 ± 7.77	11.74 ± 6.65	8.15	< 0.01	1.70	5.65	4.21 ~ 7.09					
Language	Control	22	22.82 ± 4.69	22.23 ± 4.92	2.03	0.06	0.43	0.59	−0.02 to approximately 1.20	−2.54	0.02	0.76	−4.01	−7.20 to approximately −0.82
	Study	23	21.13 ± 6.25	18.22 ± 5.64	5.58	< 0.01	1.16	2 0.91	1.83 ~ 4.00					
Social and self-help	Control	22	15.14 ± 3.98	15.00 ± 3.78	0.65	0.53	0.14	0.14	−0.30 to approximately 0.58	−2.23	0.03	0.66	−2.22	−4.23 to approximately −0.21
	Study	23	14.78 ± 3.38	12.78 ± 2.86	3.18	< 0.01	0.66	2.00	0.70 ~ 3.30					

Note: This table presents pre-intervention and post-intervention scores of the ABC total score and subscale scores (Sensory, Relating, Body and Object, Language, Social and Self-help) for ASD in the fine motor-oriented sports play group (Study) and traditional SI group (Control). Intra-group and inter-group t-tests, and effect sizes were used to compare outcomes, illustrating differences in intervention efficacy between the two groups across distinct symptom dimensions

*Abbreviations*: *ABC* Autism Behavior Checklist, *MD* Mean Difference, *95%CI* 95% Confidence Interval

Column explanations: Dependent *T* = within-group *t*-test (pre-test vs. post-test); Independent *T* = between-group *t*-test (post-intervention scores: Control vs. Study group)

## Discussion

The core symptoms of ASD are mainly social communication difficulties, stereotyped behaviors, and narrow interests, and previous clinical monitoring has mostly focused on the development of social communication ability, language, and intelligence. In recent years, more and more studies have focused on the development of motor function in children with ASD [18]; as an important indicator for monitoring children’s developmental milestones, motor function is related to the sensation, cognition, language, communication, social skills, and self-care of children with ASD. There are even studies that directly attribute stereotyped behaviors to specific types of movement disorders [19]. Moreover, studies have provided neuroimaging evidence that stereotypical behaviors and motor deficits in children with ASD share a common neural basis, including basal ganglia, cerebellum, and related cortical circuits [20], suggesting that the core symptoms of children with ASD are closely related to their motor function. Besides the core symptoms, we should also pay attention to the motor function of children with ASD. In terms of gross motor function, it is of great necessity for children with autism to have good social communication skills and normal behavior patterns when performing various gross motor tasks [21]. On the other hand, gross motor function precedes social and communication skills, the impairments of which will limit the interaction and communication between ASD children and others [22]. Similarly, deficits in fine motor function also commonly co-occur with the core symptoms of ASD in children [23]. Converging evidence from the literature and our own preliminary exploration indicated a significant negative correlation between the core symptoms of ASD and fine motor function [24]. Our study strengthens the internal validity of this association by conducting multidimensional assessments of both core symptoms and fine motor function within the same cohort. More importantly, it suggested that fine motor function represents a critical and interrelated domain in ASD, warranting inclusion in both assessment and intervention frameworks.

The correlation analysis was conducted during the research design stage based on baseline data and existing literature, and it provided a reasonable theoretical basis for our intervention design. We hypothesized that a targeted improvement in fine motor skills might yield benefits for core symptoms. We, therefore, designed a randomized controlled trial comparing fine motor-oriented sports games against traditional SI. Sensory integration therapy, which focuses on modulating tactile, proprioceptive, and vestibular inputs, is a widely used non-invasive treatment approach. Previous studies have demonstrated its positive effects in ameliorating symptoms associated with ASD [25, 26]. However, our experimental intervention was specifically tailored based on the fine motor-core symptom correlation. The sports games were engineered to embed fine motor skill challenges (e.g., precise grasping, in-hand manipulation, hand–eye coordination within tasks like bead-threading and ball-catching games) into motivating, socially interactive play contexts. The final trial demonstrated that a 12-week, fine motor-oriented sports game program significantly improved both core autism symptoms (as measured by the ABC total score) and fine motor function in children with ASD, compared to traditional SI. This dual improvement reinforces the close link between motor and social-communicative domains in ASD. Crucially, our study extends beyond merely replicating this known association; it provides novel experimental evidence that a targeted, game-based motor intervention may serve as an effective vehicle for addressing core symptoms.

The most crucial objective of this study is to explore, through a randomized controlled trial, the effects of sports game training focused on fine motor skills and traditional SI on the core symptoms of children with ASD. Undoubtedly, both intervention methods can bring about positive changes, but the sports game intervention group demonstrated more significant improvements in key indicators. SI is based on the theory of neuroplasticity, proposing that structured activities regulating vestibular, proprioceptive, and tactile input can improve the brain's processing of sensory information. This helps alleviate symptoms such as anxiety and stereotyped behaviors caused by abnormal sensory processing while also laying the foundation for higher-level social and learning functions [27]. This study supports this perspective: children in the control group showed a decrease in their total ABC score, primarily driven by changes in sensory and motor factors. This indicates that SI can effectively reduce problematic behaviors in children with ASD by improving sensorimotor integration. However, this study also found that SI has limited effectiveness in promoting social communication and self-care skills in children with ASD. This aligns with existing research, which suggests that while SI can enhance basic sensory processing and hand–eye coordination [28], its relatively structured, one-on-one activity format lacks natural embedding in social contexts, thereby limiting its generalization to social domains.

In contrast, the fine motor function-oriented sports game intervention demonstrates key differences in both its mechanisms and application, which may explain its superior efficacy: On the one hand, in terms of intervention mechanism, sports games go beyond the modulation of a single sensory channel. By embedding targeted practice within enjoyable and challenging game contexts—such as ball throwing and catching or bead threading—they naturally integrate goal-directed exercise, social reinforcement, and cognitive engagement. Completing these game-based tasks requires children to simultaneously mobilize attention, motor planning, immediate feedback processing, and cooperation. This directly engages the prefrontal-cerebellar-basal ganglia circuit, which is closely associated with executive function, motor learning, and social cognition [29]. Thus, its mechanism of action originates from “task motivation” and operates through enhancing the synergistic integration of sensory, motor, and cognitive processes to comprehensively improve functioning [30]. On the other hand, in terms of clinical outcomes, this integrative mechanism leads to broader benefits. The experimental group showed a reduction of 10–20 points in the total ABC score, which was not only statistically significant but also reached the threshold for clinically meaningful improvement. The literature suggests that a reduction of approximately 25% from baseline on the ABC-Ⅰ scale is generally regarded as clinically significant [31]. Although there is no universally accepted MCID for ABC in children with ASD in the literature yet, considering that the total score of ABC scale, this reduction is quite considerable. More importantly, the intervention effects were evident across multiple dimensions: understanding rules and taking turns during games enhanced social initiative; the sense of accomplishment from task completion reduced anxiety and withdrawal behaviors; and physiological fatigue induced by highly repetitive actions such as jumping and grasping may serve as one immediate mechanism for reducing stereotyped behaviors [32].

Our findings suggest that the sports game-based intervention should not be viewed merely as a replacement for traditional SI training, but rather as a complementary or advanced-tier option within a comprehensive therapeutic plan. For children with ASD who primarily exhibit deficits in sensory modulation, traditional SI remains a foundational and effective approach. However, for children whose challenges extend more prominently into fine motor coordination, goal-directed play, and social initiation, this sports game program could be prioritized or integrated to address these specific domains [33]. Regarding feasibility, it utilizes low-cost, accessible materials compared to specialized sensory equipment. The required practitioner training is comparable to or less than that for standard sensory integration therapy, and its group-based format offers potential cost-effectiveness advantages. Furthermore, the goal-oriented sports games were designed to simulate the real scenarios’ tasks and social interactions, thereby enhancing the ecological validity of the training. This approach may facilitate the transfer of acquired skills to home and school environments, and also confirms the generalizability of the intervention effect of this study.

Finally, it is noteworthy that one study involving children with ASD using motion-input devices for sports-oriented video games did not find significant improvement in their actual fine motor skills [34]. This contrast further highlights the crucial role of intervention format. Compared to virtual training, the physical-activity-based games employed in this study—which emphasize the manipulation of real objects and immediate interpersonal interaction—may have inherent advantages in promoting the generalization of motor skills and their integration with social functions. Specifically, this program embedded goal-oriented fine motor tasks (e.g., hand–eye coordination) into enjoyable sports games that incorporate turn-taking, rules, and cooperation. This design concept aligns closely with the core view of embodied cognition theory, both of which emphasize that cognition is rooted in bodily and environmental interactive experiences [35]. The intervention directly targets the key link between sensorimotor integration and social cognition in children with ASD: by requiring children to plan, execute, and adjust movements to achieve goals within the games, it not only improved motor function but also strengthened bodily awareness and the sense of agency in a social context. Theoretically, this lays the foundation for children to understand their own and others' behavioral intentions, thereby potentially facilitating the positive transfer of motor skills to social behaviors. Therefore, compared to isolated sensory regulation or repetitive motor training, the core advantage of this intervention lies in the fact that it does not train a single skill in isolation. Instead, through embodied, goal-oriented social play, it naturally and synergistically integrates the complete process of sensory input, motor execution, and cognitive engagement. This integrative mechanism is precisely the potential reason why it may fundamentally produce positive effects on core symptoms of ASD, such as social communication. Besides, the broad age range of our sample raises the question of whether developmental stage moderates intervention response. Younger children (< 3 years) are in a period of foundational skill acquisition, which might make them more responsive to intensive, play-based motor training. Conversely, older preschoolers (≥ 3 years) have more advanced cognitive and language capacities, potentially allowing them to better understand game rules and integrate motor tasks with social cues, possibly enhancing the transfer of skills [36]. This trial was designed and powered to detect an overall effect between intervention groups, not for subgroup comparisons. Therefore, it is underpowered to conduct robust, age-stratified analyses.

A sports game intervention guided by fine motor skills, adapted to structured clinical or educational environments, can be conducted by professionals in one-on-one or group lessons. It is applicable in outpatient rehabilitation clinics, autism intervention centers, and special education schools with personalized treatment resources. It can also be used as a group treatment module in outpatient settings to enhance the intervention effect. Under safety and supervision, its core elements can also be integrated into kindergarten and inclusive pre-school sports activities, benefiting more children with development needs. The space, resources, and standardized group arrangements of these places can meet the implementation requirements of this semi-structured game intervention. While this study demonstrates the positive effects of sports-game-based training in a Chinese rehabilitation setting, certain cultural and contextual factors should be considered when interpreting and generalizing the findings. Firstly, parental perceptions may be influenced by cultural values. In contexts where structured, traditional sensory integration are highly valued, parents might initially underreport benefits from a play-based intervention. Secondly, a detailed, standardized manual enhances replicability, yet therapists must be trained to adapt activities sensitively within their specific socio-cultural setting. Thirdly, the intervention's core principle—using intrinsically motivating play to target precise functions—may hold strong cross-cultural promise due to the universal appeal of play. Its relatively low cost and minimal equipment requirements enhance its potential transferability to diverse educational or healthcare systems with limited resources. However, successful implementation abroad would likely require careful cultural adaptation of specific game contents to align with local children's interests and social norms.

While our study further enriches the treatment options available to clinicians, the interpretation of the intervention effects requires caution and should incorporate considerations of the study’s limitations. First, the intervention duration was three months. Although positive changes were observed, the long-term sustainability of these effects remains uncertain. While the reduction in the total ABC score is multifaceted in significance, the improvement patterns in other core symptom measures (e.g., CARS) were inconsistent. This suggests that the intervention’s effectiveness may be domain-specific, and future research is needed to clarify the symptom dimensions for which it is most effective. Second, while recruiting participants from a special education institution and employing a “one-to-one” intervention model helped control for major concurrent formal therapies within that setting, we cannot entirely rule out the influence of variations in daily home activities on the outcomes. This is a common limitation in behavioral intervention studies. Although the therapist reported that the overall participation and tolerance of the children in both groups were good during the intervention process, we did not use standardized and quantitative tools to continuously measure the participation level of each treatment session. Third, while outcome assessors and participants’ parents were blinded to group allocation, the therapists delivering the interventions were necessarily aware of the treatment condition. This could introduce expectancy effects or differential therapist enthusiasm, potentially influencing outcomes. We attempted to mitigate this through rigorous therapist training, standardized protocols, and close supervision, but it remains a possible source of bias. Furthermore, this study did not conduct a formal inter-rater reliability test, which is also a limitation of this research.

## Conclusions and prospects

Based on preliminary observations indicating an association between core symptoms and fine motor function, we designed and conducted this randomized controlled trial to directly compare the efficacy of the two interventions. This approach completes a translational research cycle from exploratory correlation to experimental verification. The trial results demonstrate that a structured sports-game-based intervention focused on fine motor skills is more effective than SI in improving both the core symptoms and fine motor function of children with ASD. Our study contributes a unique perspective to the field of motor intervention: targeting fine motor skills within an engaging game-based framework can yield broader positive effects on social communication and behavioral symptoms. This suggests that embedding targeted tasks within embodied, socially interactive play contexts represents an effective intervention pathway distinct from traditional isolated training methods. Future research should focus on the following aspects: First, utilizing neuroimaging and other technologies to investigate the specific mechanisms underlying the changes in brain network connectivity induced by such interventions, particularly the synergistic patterns between sensorimotor cortex and social brain regions. Second, optimizing the exercise prescription to define the optimal intensity, frequency, and duration of the intervention, thereby establishing a more precise dose-effect relationship. Third, although this study provided empirical evidence for the overall efficacy of the intervention through comprehensive age analysis, future research with a larger sample size is still needed to clarify whether there are differences in the efficacy responses of children at different developmental stages to this intervention. At the same time, longer-term follow-up studies should also be implemented to further evaluate the persistence of the intervention effect and its impact on children's long-term social adaptability.

## Supplementary Information

Supplementary Material 1.

## Abbreviations

ABC: Autism Behavior Checklist
CABS: Childhood Autism Behavior Scale
CARS: Childhood Autism Rating Scale
Grasping: Fine motor grasping skill score
Visual-motor integration: Visual-motor integration skill score
FMQ: Fine motor quotient
MD: Mean Difference
95%CI: 95% Confidence interval
