# Supplementary material for: Effects of fine motor-skill oriented sports games on core symptoms in children with autism: a randomized controlled trial
Source: Trials. 2026 Mar 21;27:333. doi: 10.1186/s13063-026-09651-1 (PMC13130581; doi:10.1186/s13063-026-09651-1)
Supplement: Supplementary file 1 — Supplementary Material 1. [file 13063_2026_9651_MOESM1_ESM.doc]

**Supplementary Material: Detailed Intervention Protocol**

**1. Therapist Qualifications & Training**

1.1. Prerequisite Qualifications:

All therapists conducting the interventions must meet the following minimum criteria:①Hold a recognized certification in Occupational Therapy, Physical Therapy, or Special Education.②Have a minimum of one year of documented clinical experience working directly with children with Autism Spectrum Disorder (ASD).

1.2. Pre-Study Training Program:

All therapists participated in a standardized training program led by the principal investigator and a senior occupational therapist. The curriculum included:

• Theoretical Rationale: Overview of ASD core symptoms, principles of sensory integration, fine motor skill development, and the specific aims of each intervention arm.

• Protocol Mastery: Step-by-step rehearsal of all session scripts for both the treatment and control groups. Emphasis was placed on:①Delivering standardized, scripted instructions；②Correctly setting up and using all equipment；③Applying the pre-defined rules for prompting, assistance, and safety；④Implementing the scheduled progression (e.g., changing distance) at Weeks 1, 5, and 9；

• Child Engagement & Safety: Strategies for maintaining child motivation, managing behavioral challenges, and ensuring physical safety during all activities.

**2.Equipment List**

Table S1: Comprehensive Equipment List for Both Intervention Groups

| **Item** | **Specification** | **Primary Use**  **in Treatment Group** | **Primary Use**  **in Control Group** |
| --- | --- | --- | --- |
| **Mini Trampoline** | **Diameter: 90~110cm** | **Pass and Catch:Base station for jumping and catching activity.** | **Trampoline Training: Platform for vertical jumps.** |
| **Soft Foam Ball** | **Diameter: 15cm ; 10cm** | **Pass and Catch: Throwing and catching target.** | **Not used.** |
| **Balance Board** | **Different heights: 10cm ; 15cm** | **Stringing Beads: Unstable surface for balance challenge.** | **Balancing Board Training: Platform for prone rocking.** |
| **Wooden Beads** | **Diameter: 3cm ; 2cm** | **Stringing Beads: Fine motor manipulation objects.** | **Not used.** |
| **Stiff Shoelace** | **with taped end** | **Stringing Beads: String for bead threading.** | **Not used.** |
| **Therapy Swing** | **Seat with back support** | **Sharp Eyes and Agile Hand: Seated base for swinging and throwing.** | **Swing Play: Equipment for rotational vestibular input.** |
| **Rubber Loops** | **Diameter: ~20cm** | **Sharp Eyes and Agile Hand: Throwing rings for targeting.** | **Not used.** |
| **Target Pole** | **stable weighted base** | **Sharp Eyes and Agile Hand: Target for ring throwing.** | **Not used.** |
| **Target Pole** | **stable weighted base** | **Timing activity blocks, rests, and intervals for all activities.** | **Timing sets, intervals, and rotation counts** |

****3. Session Scripts****

General Session Structure (Identical for Both Groups):

Total Duration: 30 minutes.

Warm-up: 5 minutes of guided, low-intensity gross motor movements (e.g., arm circles, marching in place).

Core Activity Blocks: Three blocks of 7 minutes each, dedicated to the three core activities.

Rest Intervals: 2 minutes of seated, quiet rest between blocks.

Scripted Instruction: Therapists must use the phrases in quotes verbatim.

Table S2: Treatment Group Protocol - Weekly Session Script (Example: Week 1)

| **Activity** | **Primary Objective** | **Step-by-Step Procedure & Scripted Instruction** | **Progression Rules** |
| --- | --- | --- | --- |
| **Pass and Catch** | **Dynamic hand-eye coordination, bilateral catch.** | **1.Child stands on mini-trampoline. Therapist stands 1.0m away；2.Therapist throws 15cm ball gently toward child's torso； 3.Instruction:“[Child’s name], keep jumping! Catch the ball and throw it back to me.” 4.Child attempts to catch with two hands and throw back.** | **At Week 5,distance increases to 1.5m；**  **At Week 9, ball size decreases to 10cm.** |
| **Stringing Beads on Balancing Board** | **balance function+ precision fine motor** | **1.Child stands on 10cm-high balance board.**  **2. Beads and lace are placed on a table within arm's reach.3. Instruction: “Try to stand steady. Pick up one bead and put it on the string. Now try the other side.”4. Child strings beads while maintaining balance.** | **At Week 5, board height to 15cm.**  **At Week 9, bead size to 2cm.** |
| **Sharp Eyes and Agile Hand** | **Visual tracking & timing, proximal stability, aiming.** | **1. Child sits on swing. Target pole placed 1.5m ahead.2. Therapist gives a gentle, predictable push to create a steady arc.3. At forward peak, child throws a rubber loop at the pole.4. Instruction: “When you swing forward, try to ring the pole!”** | **At Week 9, therapist provides gentle,unpredictable pushes to vary rhythm.** |

Table S3:Control Group Protocol - Weekly Session Script (Example: Week 1)

| **Activity** | **Primary Objective** | **Step-by-Step Procedure & Scripted Instruction** | **Progression Rules** |
| --- | --- | --- | --- |
| **Trampoline Training** | **Vestibular-proprioceptive input, motor endurance.** | **1.Child performs continuous vertical jumps, feet together.2.Instruction:“Jump up and down… landing with both feet together.”3. Structure:3 sets of 10 jumps, with 30-second seated rest between sets.** | **At Week 5, add knee tucks mid-jump.**  **At Week 9, add coordinated arm swings.** |
| **Balancing Board Training** | **Proprioceptive awareness, core strength** | **1.Child lies prone on board, gripping handholds.2. Rocks board side-to-side using trunk.3.Instruction:“Hold on tight… rock the board side to side like a seesaw.”4. Structure:4 intervals of 45s rocking / 45s rest.** | **At Week 5, increase amplitude.**  **At Week 9, perform task with eyes closed.** |
| **Swing Play** | **Vestibular adaptation, postural control.** | **1. Child sits in therapy swing.2.Therapist applies fixed rotation sequence:10 rotations clockwise, pause, 10 rotations counter-clockwise. Repeat twice. 3.Total:~7-8 minutes of swinging.** | **At Week 5, slightly increase rotation speed. At Week 9, introduce brief,unexpected pauses (2-3 sec) during rotation.** |

**Note on Progression: The protocol includes scheduled difficulty increases. All changes (distance, size, height, task variant) are implemented at the start of the week indicated and maintained thereafter.**

**4. Safety & Contingency Guidelines**

A spotter must be within arm’s reach for all balancing and swinging activities.

Sessions must be conducted over safety matting.

If a child shows signs of distress (e.g., crying, covering ears, attempting to leave), the activity should be paused immediately. The therapist may offer a simplified version or, if necessary, move to the next activity after a calming period.

All equipment must be inspected for damage before each session.
